# Structural Properties of PAS Domains from the KCNH Potassium Channels

**DOI:** 10.1371/journal.pone.0059265

**Published:** 2013-03-15

**Authors:** Ricardo Adaixo, Carol A. Harley, Artur F. Castro-Rodrigues, João H. Morais-Cabral

**Affiliations:** IBMC, Instituto de Biologia Molecular e Celular, Universidade do Porto, Porto, Portugal; Virginia Commonwealth University, United States of America

## Abstract

KCNH channels form an important family of voltage gated potassium channels. These channels include a N-terminal Per-Arnt-Sim (PAS) domain with unknown function. In other proteins PAS domains are implicated in cellular responses to environmental queues through small molecule binding or involvement in signaling cascades. To better understand their role we characterized the structural properties of several channel PAS domains. We determined high resolution structures of PAS domains from the mouse EAG (mEAG), drosophila ELK (dELK) and human ERG (hERG) channels and also of the hERG domain without the first nine amino acids. We analyzed these structures for features connected to ligand binding and signaling in other PAS domains. In particular, we have found cavities in the hERG and mEAG structures that share similarities with the ligand binding sites from other PAS domains. These cavities are lined by polar and apolar chemical groups and display potential flexibility in their volume. We have also found that the hydrophobic patch on the domain β-sheet is a conserved feature and appears to drive the formation of protein-protein contacts. In addition, the structures of the dELK domain and of the truncated hERG domain revealed the presence of N-terminal helices. These helices are equivalent to the helix described in the hERG NMR structures and are known to be important for channel function. Overall, these channel domains retain many of the PAS domain characteristics known to be important for cell signaling.

## Introduction

The KCNH family of voltage gated potassium channels includes the EAG (ether-a-go-go) channels, the ERG (eag related gene) channels and the ELK (eag like K^+^) channels [1]; these channels have roles in cardiac repolarization [2], neuronal excitability [3], cell differentiation and tumor growth [4]. A member of this family of channels, the human ERG (hERG) channel, is well known as the molecular basis of Long QT2 syndrome, a condition characterized by cardiac arrhythmias and sudden death [2].

The KCNH potassium channels have the characteristic tetrameric organization of voltage-gated channels [1]. Each subunit has six trans-membrane helices where the first four form the voltage-sensor domain and the last two, together with equivalent regions from the other three subunits, compose the channel pore. A defining characteristic of KCNH channels is their large N- and C-terminal cytoplasmic regions. The N-terminus typically includes a Per-Arnt-Sim (PAS) domain [5], while the C-terminal region includes a domain that strongly resembles cyclic nucleotide binding domains [6], [7], but which has been shown to have little affinity for cyclic nucleotides [8]. The functional role of these N- and C-terminal globular domains is unclear; however, based on the functional role of related domains in other proteins [9], [10], [11] it is reasonable to think that these domains have a regulatory role, acting as interfaces between the channel and the signaling pathways in the cell.

PAS domains are widespread in prokaryotes and eukaryotes [11], [12]. Despite a highly variable amino acid sequence the PAS domain fold is well conserved [10] PAS domains are involved in the regulation of cellular responses to environmental queues (for example: light, ligand levels, redox potential) by acting directly as a sensor domain or by participating in the response pathway [11] ^;^ [12]. The functional role of the PAS domain, in particular the direct sensing of environmental queues, depends in many instances on the interaction with a small molecule, such as the heme, flavin mononucleotide (FMN) or carboxylic acid compounds (malonate, succinate or citrate) [10], [12]. There are however, many PAS domains for which no small molecule ligands have been identified and which are considered orphan receptors. In addition, some PAS domains share a mechanistic feature where signaling involves modulation of protein-protein interactions established through the PAS domain β-sheet [10].

As already stated, the role of PAS domains in KCNH channels is unknown; it is not known if these domains bind small ligands or mediate interactions between the channel and other proteins. It has been well demonstrated that an N-terminal truncation (which includes removal of the PAS domain) of the hERG channel [13] or of the rat EAG channel [14] results in changes on the functional properties of channels, in particular gating (opening and closing of the channel). Much of this effect can be pinpointed to the very first 10–15 residues of the N-terminus, which have been shown to be disordered in the X-ray crystal structure [5] and NMR structures [15], [16], [17] of the hERG channel PAS domain. hERG channel native properties can be partially restored to an N-terminally truncated channel by addition of a peptide with the amino acid sequence corresponding to the first 16 residues of the wild-type channel [18]. Injection of isolated PAS domain protein, or co-expression of the domain, in a cell expressing N-terminally truncated channel will also restore wild-type activity [5], [19]. Importantly, it has been demonstrated that the restoration of native activity results from binding of the PAS domain polypeptide to the truncated channel [19]. The determination of the structure of the hERG PAS domain led to the identification of a hydrophobic surface on the β-sheet that is probably involved in the interaction between the domain and the rest of the channel [5]. In addition, mutations on this surface result in functional changes that are equivalent to the truncation of the whole domain [5]; some of these mutations also affect the targeting of the domain to the truncated channel and reconstitution of wild-type properties [19].

In this study we are interested in better understanding the structural properties of PAS domains in the KCNH channels. We have determined the structures of two domains, from the mouse EAG and drosophila ELK channels. We have also re-determined the structure of the PAS domain from the hERG channel at a higher resolution and determined the structure of the same domain without the first 10 amino acids. We have analyzed and compared these structures to establish the existence of potential binding sites for small molecules. We have also defined the structural features that are shared with other PAS domains, as well as the characteristics that are unique to PAS domains in channels.

## Materials and Methods

### Protein Expression and Purification

PAS domains from mouse EAG (mEAG) and *drosophila* ELK (dELK) channels were cloned, expressed and purified as previously described [20]. Briefly, domains were purified by affinitiy His-tag chromatography, followed by removal of His-tag with thrombin. Further purification was done by size-exclusion chromatography. PAS domain from dELK was methylated at lysines according to [20], [21]; the reaction was stopped by size-exclusion chromatography.

PAS domain from the human ERG domain was cloned, expressed and purified as previously described [5], [22]. hERG PAS domain starting at residue 10 (Δ9-hERG) was cloned as a BamHI/EcoRI cassette into pGEX2T plasmid. The Δ9-hERG expression and purification was essentially as described for the full length hERG PAS construct.

### Crystallization

Crystals of mEAG and dELK were obtained as previously described [20]. Briefly, mEAG crystals were obtained by mixing a mEAG PAS solution at 10 mg/ml with precipitant solution: 1.3 M ammonium sulfate, 0.25 M lithium sulfate, 0.1 M Tris–HCl pH 8.5; dELK crystals were obtained by mixing methylated dELK PAS at 10 mg/ml with precipitant solution: 0.1 M MES pH 6.5, 30%(w/v) PEG 5000 MME, 0.35 M ammonium sulfate.

Crystals of hERG PAS domain were obtained in similar conditions to the previously described [5] with the following changes: the protein solution did not contain detergent and the reducing agent included was 1 mM TCEP. In addition cryo-protection of crystal was achieved by quick transfers through crystallization reservoir solution (1.05 M Na^+^/K^+^ tartrate, 100 mM Hepes pH 7.0) containing increasing amounts of glycerol (5–20%) and 1 mM guaiaretic acid.

Initial crystallization conditions for Δ9-hERG (at 10 mg/ml in 50 mM Hepes pH 7.5; 150 mM NaCl; 10 mM DTT) involved screening at 20°C using commercial formulations. Two crystallization conditions were identified: 0.1 M potassium thiocyanate, 30% (w/v) PEG MME 2000 and 0.15 M potassium bromide, 30% (w/v) PEG MME 2000. Both conditions were optimized by fine-grid and additive screening and larger crystals were obtained at room temperature with 0.1 M potassium thiocyanate, 30% (w/v) PEG MME 2000 and 0.5–2.0% (w/v) benzamidine.HCl.

### Data Collection, Processing and Refinement

Data were collected at ID14-4 and ID14-1 (ESRF) and PXIII (SLS). Datasets were processed with Mosflm [23] or XDS [24], and SCALA [25]. Molecular replacement was performed with Phaser [26] using the hERG PAS domain (PDB code 1BYW) as a search model, model building was done in Coot [27] and refinement was performed with TLS [28] using Refmac [29] and Phenix [30]. Figures were generated with Pymol [31]. Coordinates and structure factors have been deposited in the Protein Data Bank: 4HQA (hERG PAS domain), 4HP9 (Δ9-hERG PAS domain), 4HOI (mEAG PAS domain) and 4HP4 (dELK PAS domain).

## Results

### Cavity in the PAS Domain of the hERG Channel

**Figure 1:**
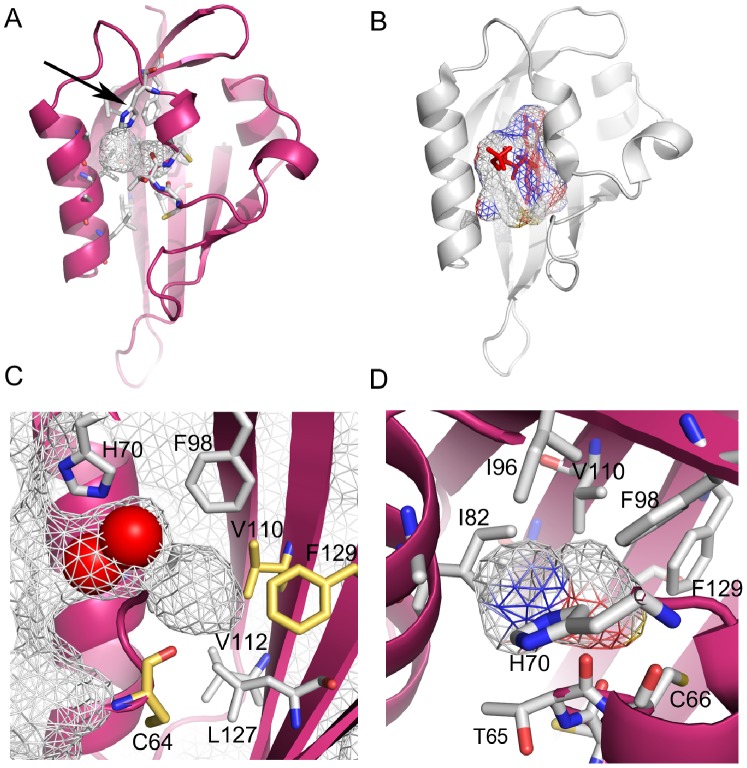
Cavity in the PAS domain of hERG channel. a) Cartoon representation of high resolution structure of PAS domain from hERG channel. Cavity is shown as wireframe surrounded by several residues shown in stick. Arrow indicates residue H70 at the entrance to cavity. b) PAS domain structure of phototropin Phot-LOV1 (PDB code 1N9N). Flavin mononucleotide, shown as red stick, is bound within binding site cavity, shown as wireframe. c) Detailed view on hERG PAS domain cavity. The molecular surface of the domain is shown as wireframe and extends to the domain cavity. Water molecules in cavity are shown as red spheres. Some of the residues lining cavity are shown and labeled. Residues that in other channel PAS domain are substituted by polar residues are shown in gold. d) Polar and apolar chemical groups lining the cavity of the hERG PAS domain are shown. Wireframe delimiting cavity is colored according to the atoms that compose the cavity lining, red for oxygen, blue for nitrogen and white for carbon; the polar face (predominantly colored red and blue) of the cavity is towards the reader and the apolar face is at the opposite side.

We have re-determined the structure of the PAS domain from the hERG channel at a higher resolution (1.96 Å) (Figure 1a and Table 1). A comparison between the new structure and the 2.6 Å structure previously reported (PDB code 1BYW) shows that they are identical (root-mean-square deviation (rmsd) for main-chain atoms of 0.33 Å). Like in the original crystal structure, the first 25 residues are not defined in the high resolution electron-density maps. Strikingly, both structures show a hollow space in the region where other PAS domains bind small molecules (Figure 1a and 1b). Analysis of the high resolution structure by the CASTp server [32] (http://sts-fw.bioengr.uic.edu/castp/calculation.php) reveals the presence of two adjacent cavities connected by a ∼2 Å wide gap, but for the purpose of this study they will be discussed as a single feature (Figure 1a and 1c). This cavity is open to the bulk solvent, does not contain any small molecule ligand but is partially occupied by 2 water molecules (Figure 1c). Its total volume is ∼110 Å^3^; it is ∼8 Å deep and has a maximum width of ∼5 Å. The cavitýs lining has a polar and an apolar face (Figure 1d). The polar face is mainly formed by chemical groups from the proteińs main-chain, including the carbonyls from C64, T65 and A79; this face also includes the side-chain from H70, which is positioned at the mouth of the cavity, and the mildly polar thiol group from C66. The hydrophobic face is lined by apolar side-chains from I82, I96, F98, V110, L127 and F129.

**Table 1:** Crystallographic data and refinement statistics.

Crystal	mEAG	dELK	hERG	Δ9-hERG
Space group	P3_2_21	P4_1_32	P6_5_22	P4_2_32
Unit cell parameters				
a = b (Å)	93.3	150.3	56.7	89.5
c (Å)	172.3	150.3	134.7	89.5
α = β (°)	90	90	90	90
γ (°)	120	90	120	90
**Diffraction statistics**				
Resolution range (Å)	43–1.85 (1.95–1.85)	61–2.00 (2.11–2.00)	33–1.96 (2.06–1.96)	40–2.12 (2.23–2.12)
No. unique reflections	71849 (10400)	39431 (5615)	9923 (1380)	7429 (1020)
No. measured reflections	536953 (57622)	577984 (39457)	165724 (23033)	73194 (7979)
Multiplicity	7.8 (5.5)	14.7 (7.0)	16.7 (16.7)	9.9 (7.9)
Completeness (%)	100 (99.9)	99.5 (99.4)	100 (100)	99.5 (99.5)
I/σI	7.5 (1.3)	6.4 (1.4)	5.6 (0.9)	8.5 (1.1)
Rsym (%)	7.0 (56.3)	8.9 (55.0)	10.3 (72.4)	8.9 (69.1)
**Refinement statistics**				
Resolution range (Å)	40–1.85 (1.87–1.85)	30–2.0 (2.05–2.00)	33–2.05 (2.35–2.05)	44.8–2.12 (2.28–2.12)
No reflections	71734	39372	9877	7403
Rwork/Rfree (%)	16.6/19.2 (24.3/23.9)	17.3/19.2 (23.3–25.9)	20.0/23.9 (23.7/25.7)	19.4/23.8 (31.4/38.6)
No of:				
Protein residues	447	249	110	119
waters	395	210	40	89
sulfates	6	2		
glycerol		1		
RMSD bond length (Å)	0.011	0.003	0.004	0.008
RMSD bond angles (°)	1.214	0.742	0.797	1.033

**Note** - values in parenthesis correspond to statistics for data in the highest resolution shell. Diffraction statistics for mEAG and dELK crystal were taken from [20].

Unlike the cavity in the hERG PAS domain, which is dominated by apolar side-chains, in PAS domains known to bind small molecule ligands, and for which structures of complexes are available, the residues forming the binding cavity quite often include polar residues [10]. Interestingly, analysis of a sequence alignment of PAS domain from KCNH channels reveals several domains where polar amino acids replace the apolar residues lining the hERG PAS cavity (Figure 1c and Figure S1). For example, in the PAS domain from the drosophila EAG-like (ELK) channel a histidine substitutes one of the phenylalanines (F129) that lines the hERG cavity, in the mouse EAG channel a serine replaces one of the hERG cysteines (C64), in other cases a threonine substitutes a hERG valine (V110). According to the structure of hERG PAS the side-chains of these polar residues are positioned within the hydrophobic core of the domain and close to the cavity seen in the hERG PAS (Figure 1c) raising the possibility that they can change the cavity properties.

### Structures of PAS Domains from Other KCNH Channels

To better understand the structural features of the cavities present within the hydrophobic core of the KCNH PAS domains we have determined the domain structures from two different channels: the mouse EAG1 (mEAG) channel and the drosophila ELK (dELK) channel. Initial crystallization trials, with domain constructs equivalent to the one defined for the hERG PAS domain structure, failed. Crystals of mEAG PAS domain were obtained by removing the first 26 residues of the domain [20], which correspond to the disordered region detected in the hERG PAS structure [5]. Crystals of dELK PAS domain were obtained after applying a strategy that involves methylation of lysines and removal of the first 10 amino acids of the protein [20]. The structures of mEAG PAS domain and dELK PAS domain were determined at 1.85 Å and 2.0 Å, respectively (Table 1).

**Figure 2:**
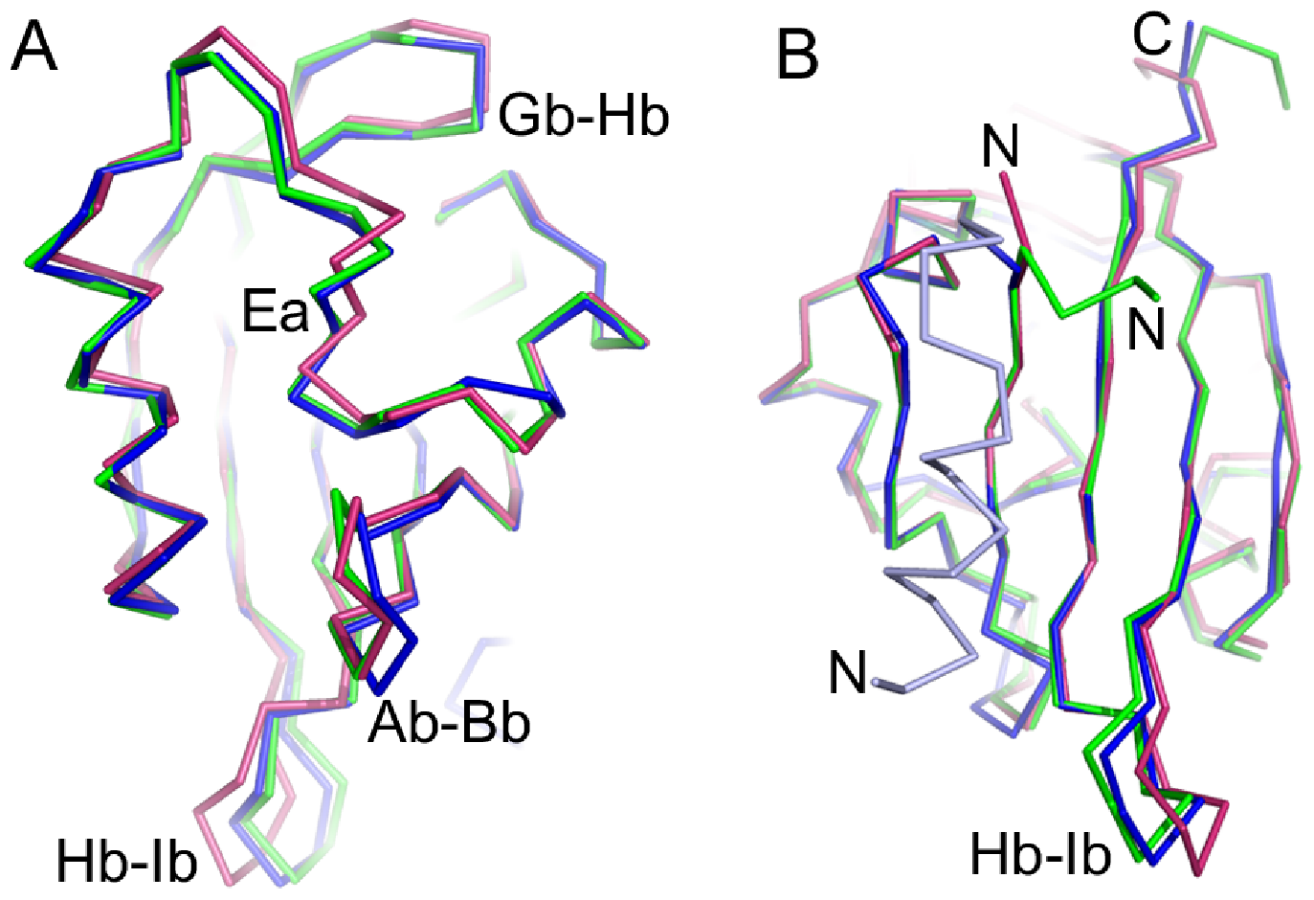
Cα representation of PAS domains from three different channels. a) View of the helical face of PAS domains. b) View of the β-sheet face of PAS domains. Colors are: hERG in redbrick, mEAG in green and dELK in blue. The N-terminal helix in dELK PAS domain (absent in the other two structures) is shown in light blue. N- and C- terminal are labeled in b). Several structural features discussed in the text are labeled.

The asymmetric unit cell of mEAG PAS domain crystals contains four copies which, excluding the N- and C-termini, are very similar to each other (rmsd for main-chain atoms of residues 28 to132 varying between 0.2 and 0.6 Å). In the dELK PAS domain crystals, the asymmetric unit contains two very similar copies (rmsd 0.4 Å for main chain atoms of all residues). Overall, the PAS domains of hERG, dELK and mEAG show a strong structural similarity (Figure 2), with a rmsd for main-chain atoms (with Herg residues 27–131 as reference) of 0.8–0.9 Å. Superposition of the domains through main-chain atoms in the β-sheet (Figure 2) shows that the major differences occur in the loop regions, in particular in loops between strands Aβ-Bβ, Gβ-Hβ and Hβ-Iβ and also in the N- and C-termini. There are also differences in the Eα 3_10_ helix and adjacent regions; the conformation of this whole stretch is similar across the three domains but the stretch is a repositioned in mEAG and dELK relative to hERG. The dELK structure also includes an N-terminal helix packed against the domain β-sheet (discussed below).

**Figure 3:**
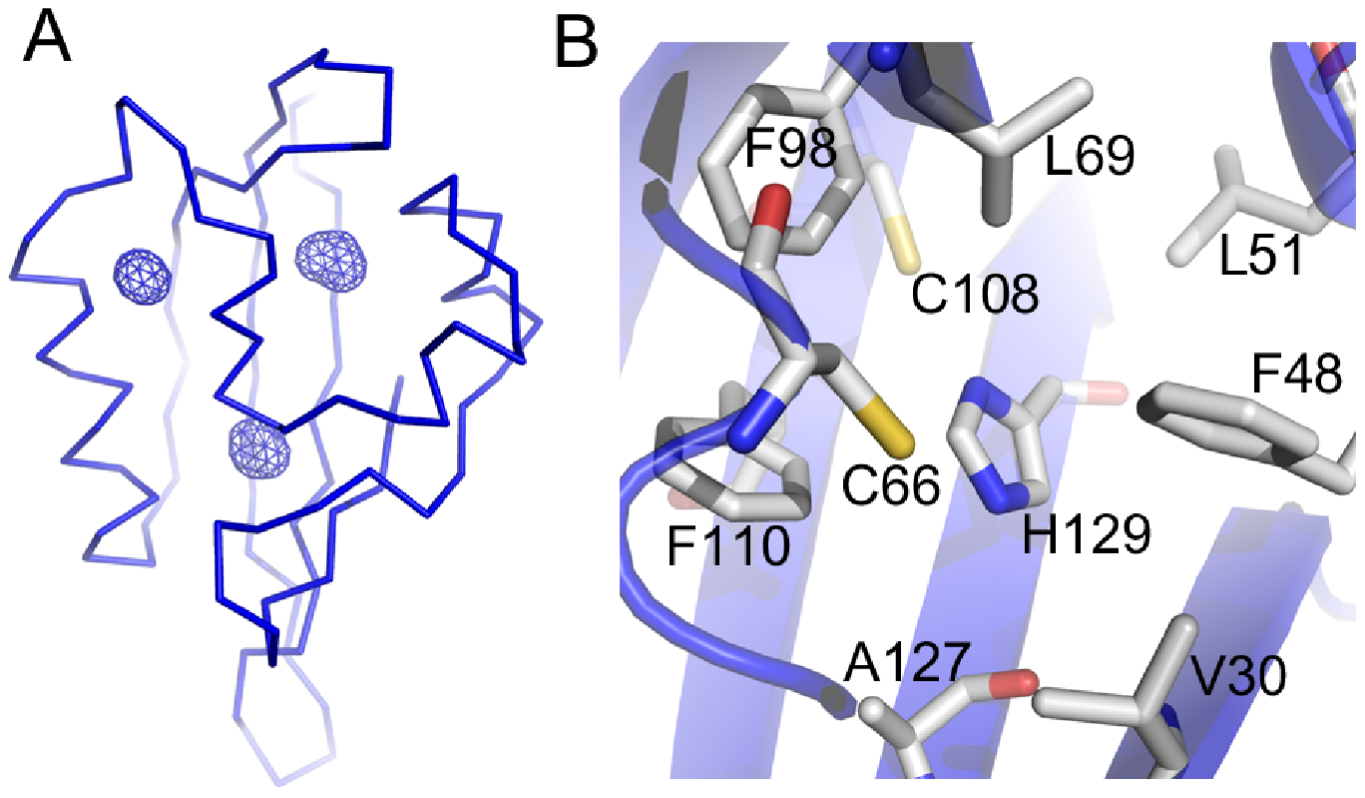
Cavities in the PAS domain from dELK channel. a) Cα representation of structure of domain. Cavities detected by CASTp server are shown as blue wireframe representations. b) Histidine 129 and surrounding residues.

Analysis of the two dELK structures in the asymmetric unit reveals the absence of a large cavity. However, residue (C64) in the hydrophobic core of the structures displays two different side-chain conformations; to account for the possible influence of this flexibility on the volume of any potential cavities, we generated two different models (each with a unique side-chain conformation) for each structure in the asymmetric unit and calculated the volume of cavities by CASTp. The CASTp server analysis revealed three very small cavities in the core of the protein of one of the generated models (with volumes 14, 15 and 23 Å^3^) (Figure 3a), while in the other 3 models, there were either two of the cavities (the 14 and 15 Å^3^), one (23 Å^3^) or none. The cavities detected in dELK do not overlap with the cavity in hERG and, importantly, their small size and apparent instability (the cavities appear and disappear in the different models) lead us to consider that they are not a conserved feature and not related to the binding sites seen in other PAS domains. In support of this conclusion, a recent analysis [33] of the structural properties of protein domains using CASTp has shown that the largest group of cavities detected in proteins (50% of total) consists of the very small ones, with volumes between 10–20 Å^3^, which most likely reflect small packing defects in the core of the structures. The lack of a preformed cavity in the dELK domain, at the same site as in hERG, appears to result from the presence of several large side-chains in that region (Figure S2). A large part of the volume is occupied by the side-chain of F110 in dELK, which substitutes the smaller valine residue present in hERG (V110). There are also adjustments in the positions of the main-chains of F98 (corresponding to F98 in hERG), of C66 (C66 in hERG) and of I82 (I82 in hERG) which contribute for the disappearance of the cavity in dELK.

As discussed above, in dELK a histidine (H129) replaces the cavity lining phenylalanine present in hERG (F129). The absence of a cavity in dELK leaves the histidine side-chain buried in the domain hydrophobic core surrounded, within 5 Å, by the side-chains of several apolar residues (V30, F48, L51, L69,F98, F110 and A127) and two cysteines (C66 and C108) (Figure 3b). The stability of this arrangement may result from hydrogen bonding by either the thiol groups of the two cysteines (at 3.5–4.0 Å from atoms in the histidine imidazole group) or by main-chain polar groups from neighboring residues (positioned at shorter distances but at less favorable angles for hydrogen bonding) or by a potential cation-π interaction between the histidine side-chain and the aromatic ring of F48 (Figure 3b).

**Figure 4:**
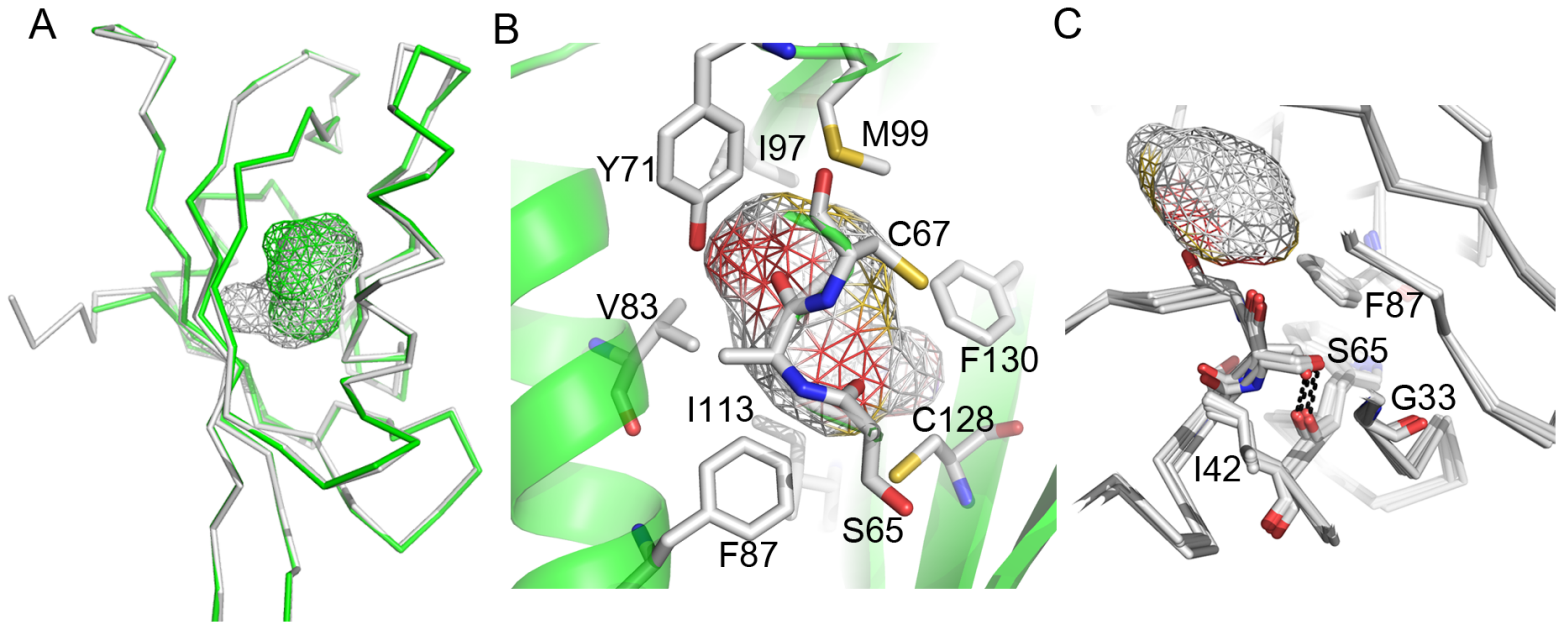
Cavities in the PAS domain from the mEAG channel. a) Cα representations of two of the models (in green and gray) generated by combinations of side-chain conformers present in the asymmetric unit molecules of the mEAG PAS domain crystal. The cavities shown in gray and green wireframe correspond to the largest and smallest cavities, respectively, detected by CASTp server. b) Residues lining the largest cavity are shown as stick and are labeled. Cavity represented as wireframe. Colors on wireframe correspond to atoms lining the cavity: red for oxygens, yellow for sulfurs, white for carbons. The face of the cavity away from the reader is apolar and only colored white. c) Superposition of the 4 molecules in the asymmetric unit of mEAG PAS domain crystal. Serine in the hydrophobic core (S65) and surrounding residues are shown in stick, cavity is shown as wireframe.

In contrast to dELK, all four structures in the asymmetric unit of the mEAG PAS domain show cavities that overlap with the one detected in hERG (Figure 4a). The high resolution mEAG domain structures reveal multiple conformations for some side-chains, in particular in residues surrounding the cavity (S65, T86, I97, F109 and V111). To account for the influence of this flexibility on the volume of the cavities, we generated models with all the combinations of conformers detected in each asymmetric unit molecule. Strikingly, the volume of the cavity in the mEAG PAS domain varies among the different models generated, from a minimum of ∼111 Å^3^ to a maximum of ∼136 Å^3^ (Figure 4a); the largest cavity has as maximum dimensions ∼8 6.5 6 Å. Only the conformers of V111 have any effect on the cavity properties, the remainder of the variation comes from adjustments in main-chain atoms. The variation in the cavity volume reveals flexibility within the core of this PAS domain which is reflected in the properties of its cavity.

Unlike in hERG, the cavity in mEAG is isolated from the bulk solvent by a lid formed by a tyrosine side-chain (Y71) (Figure 4b); this residue occupies the same position as H70 in hERG (at the mouth of the cavity) but its larger side-chain occludes the opening. Like in hERG, the mEAG cavity has both polar and apolar faces (Figure 4b). The residues that line the cavity defined in each conformer model vary but, in general, the polar face is determined by main-chain carbonyl groups from residues S65 and A66, as well as by the hydroxyl of the side-chain from Y71 and thiol groups from C67 and C128. The apolar face is formed by side-chains of apolar residues: V83, F87, I97, M99, V111, I113, and F130. No density for water molecules was detected in the cavities of any of the molecules in the asymmetric unit.

The mEAG PAS serine (S65), which replaces hERG C64 in the core of the domain, is positioned near the cavity region and the serine main-chain carbonyl group participates on the cavity lining (Figure 4c). However, in all conformer models generated for mEAG the serine side-chain does not participate in the lining of the cavity. Instead, the side-chain hydroxyl group is within hydrogen bonding distance (2.7–2.8 Å) of the main-chain carbonyl from residue W40 and does not appear to affect packing in the core of the domain.

### Surface Hydrophobic Patch and the N-terminus of the Channel PAS Domains

There are also two other regions of the hERG PAS domain that are of functional interest and which were analyzed in the new structures: the hydrophobic patch on the surface of the β-sheet and the N-terminus of the channel. The hydrophobic patch on the β-sheet of the hERG PAS domain is thought to mediate interactions between the domain and other regions of the channel, while the N-terminus (∼20 residues) has been shown to be crucial for determining the characteristically slow deactivation (closing of the channel gate) seen in the hERG channel.

**Figure 5:**
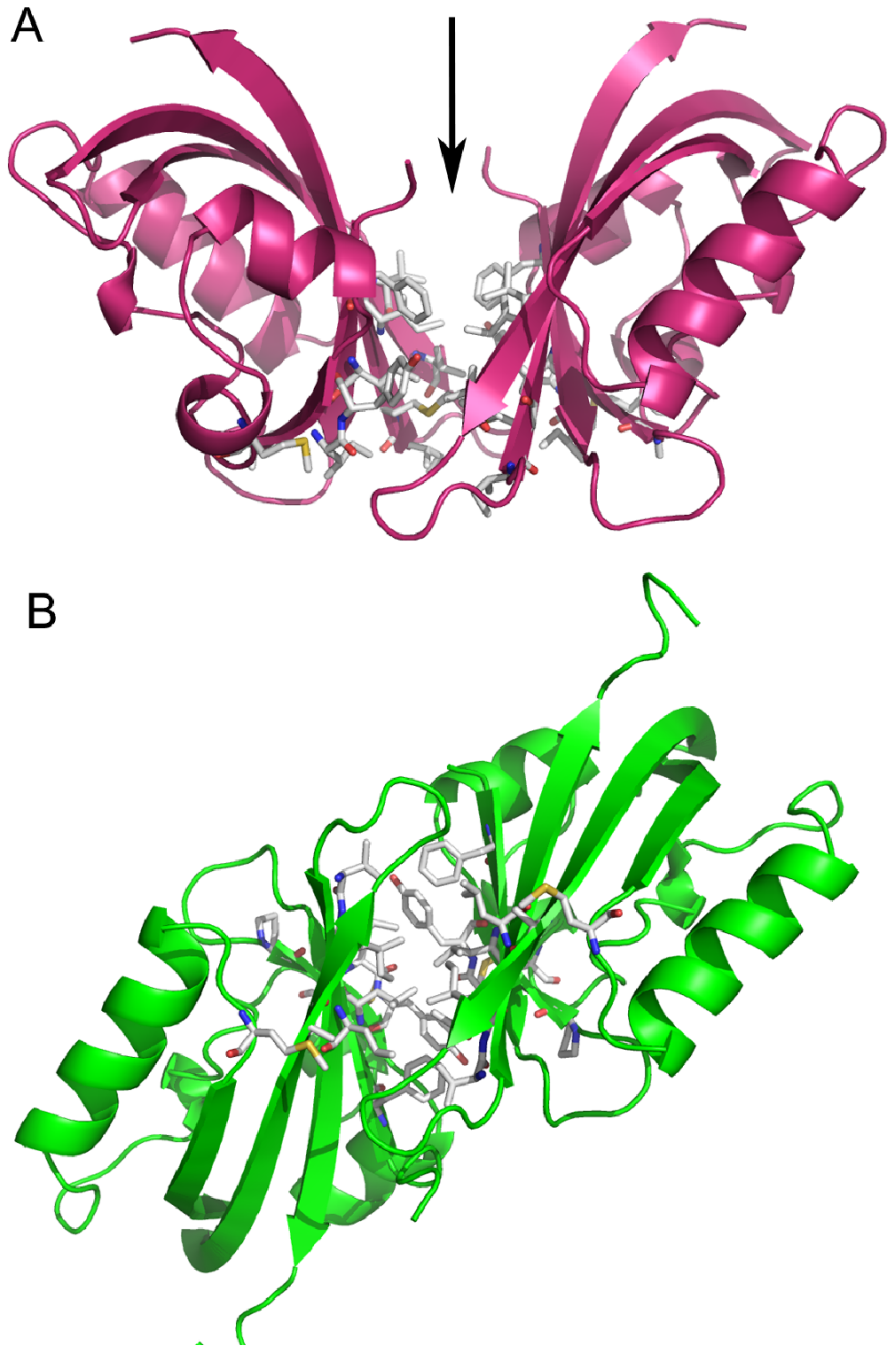
Crystal contacts formed by hydrophobic patch. a) Lattice packing of two molecules of hERG PAS domain. The molecules are related by 2 fold crystallographic axis indicated by arrow. b) Packing of two molecules in the asymmetric unit of the mEAG PAS domain. The two molecules have a ∼2-fold relation that is roughly perpendicular to page. In both panels the two molecules on the right are shown in a similar orientation. Residues from the hydrophobic patches are shown as stick.

The hydrophobic patch on the β-sheet of the hERG PAS domain includes 11 residues (Figure S3a). This patch is present in both the mEAG and dELK PAS domains and, with the exception of an extra residue in mEAG, all the same positions are occupied by apolar residues (Figures S3b and S3c). The patch mediates an extensive lattice contact in both the hERG and the mEAG PAS crystals (Figure 5); in these crystals a patch-to-patch interaction between non-crystallographically or crystallographically related protein neighbors buries a total surface area of ∼1300 Å^2^ in hERG and ∼1500 Å^2^ in mEAG. In hERG two domains pack almost face-to-face through their β-sheets (Figure 5a). In mEAG the packing arrangement is different, with the β-sheets of the paired domains inverted relative to each other (Figure 5b).Despite the extensive surface area involved in these interactions, the variability in the packing arrangements observed in the hERG and mEAG crystal lattices suggests that these domain-to-domain contacts are not structurally relevant for the architecture of the full-length channel, which contrasts with the homo- or hetero-dimeric PAS domain organizations seen with some other proteins [10].

**Figure 6:**
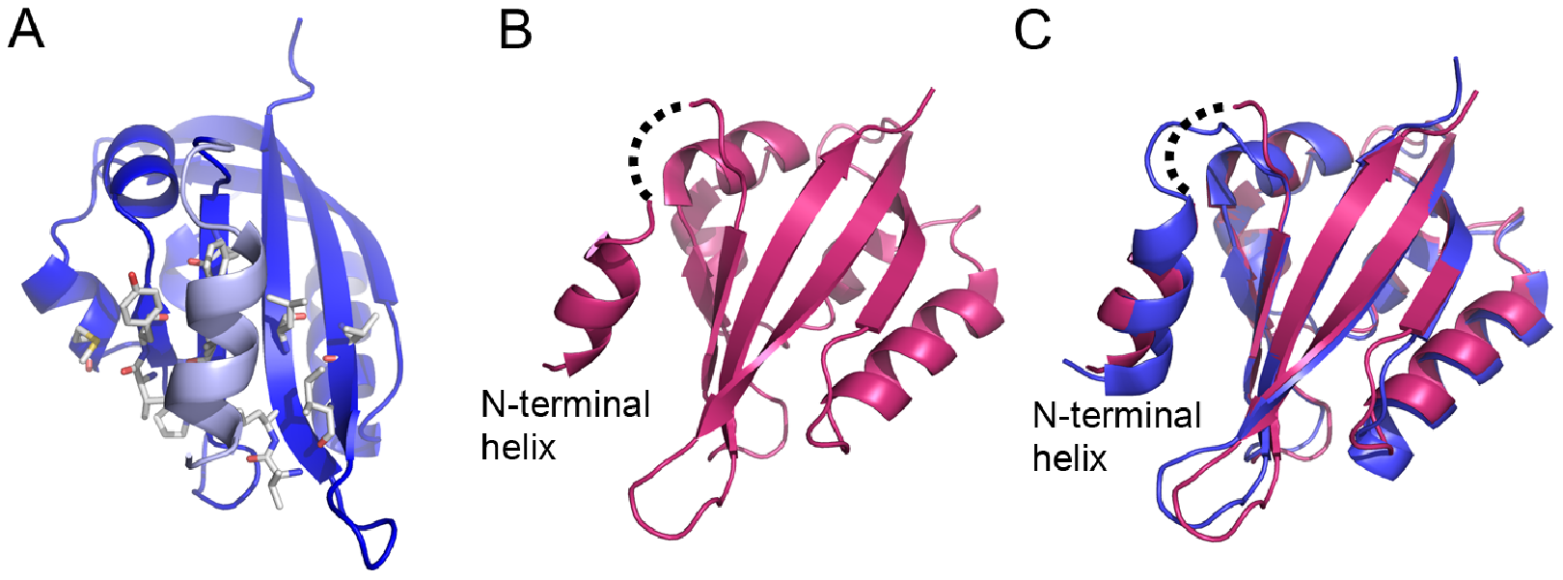
N-terminal helices in channel PAS domain structures. a) View of N-terminal helix (light blue) packed against hydrophobic patch of dELK PAS domain. Residues forming the patch are shown as stick. b) View of Δ9-hERG PAS structure with N-terminal helix packed against the β-sheet. Dotted line represent a possible linker connection between N-terminal helix and the body of the Δ9-hERG PAS domain. c) Superposition of structures of Δ9-hERG PAS (redbrick) and dELK (blue). N-terminal helices are indicated. Dotted line as in b).

In contrast, in dELK the hydrophobic patch is not involved in crystal lattice packing. Instead part of the domain N-terminus forms an amphipathic α-helix (residues 14 to 21) which packs its apolar face against the hydrophobic patch on the surface of the β-sheet (Figure 6a and Figure S3d). While the mEAG PAS construct does not include any of the N-terminal residues present in dELK, in the hERG construct the whole of the N-terminus (amino acids 1 to 25) is present but these residues are disordered in the crystal lattice [5]. The presence of a well ordered N-terminal helix in the crystal structure of dELK and its absence in hERG is intriguing since several recent NMR structures of the hERG PAS domain revealed that part of the N-terminal region (roughly residues 15 to 22) adopt a helical conformation [15], [16], [17], while residues 1–15 do not adopt a defined structure. In these NMR structures the position of N-terminal helix is not fixed and it does not establish interactions with other regions of the domain.

Based on these observations we generated a construct of the hERG PAS domain that started at residue 10, the Δ9-hERG PAS; this protein was crystallized and we solved its structure at 2.12 Å resolution (Table 1). While the body of the domain in the Δ9-hERG PAS is identical to the full-length PAS structure, with a main-chain atom rmsd of 0.74 Å (residues 26–135), the N-terminus adopts a new conformation, with residues 13 to 22 forming a helix that packs against the hydrophobic patch on the domain (Figure 6b). A consequence of the packing of the helix against the domain is that it prevents the participation of the hydrophobic patch in crystal lattice contacts. Some of the amino acid side-chains in this N-terminal helix are not well defined in the electron-density; similarly, two residues (E23 and G24) in the linker connecting the helix to the first β-strand of the domain are not defined and it is therefore not possible to determine if the N-terminal helix packs against its own domain or is swapped with a neighbor in the crystal lattice (Figure 6b). Interestingly, a superposition of the dELK structure with the Δ9-hERG PAS domain through residues in their β-sheet reveals that the N-terminal helices occupy the same relative positions at the center of the hydrophobic patch of each domain (Figure 6c). These results reinforce the NMR structural data for the formation of an amphipathic helix in part of the N-terminus of the PAS domain. They also support the idea that the crystallographic disorder detected for this region in the full-length hERG PAS domain structure is probably a consequence of the entropic penalty of restricting the position of the first 9 residues which are highly disordered in the NMR structures.

## Discussion and Conclusions

A defining feature of KCNH channels is the presence of N-terminal PAS domains. However, despite many years of functional studies, the role of these domains remains unclear. In this study we have determined the structures of the PAS domains from the drosophila ELK and mouse EAG channels and also a structure of the hERG PAS domain adopting a new conformation. These structures have allowed us to solidify our understanding of the structural properties of PAS domains in K^+^ channels.

Our analysis of the hERG, dELK and mEAG PAS domain structures revealed strong parallels with other functionally better characterized PAS domains. In many PAS domains the β-sheet mediates interactions, through a patch of hydrophobic residues, with either other PAS domain β-sheets or with non-PAS domain regions [10]. These interactions play in many cases crucial functional roles [34], [35], [36]. The hydrophobic patch on the β-sheet of channel PAS domains is a strongly conserved feature and its position, size and apolar chemical character resembles the patches seen in PAS domains from other proteins. It is also clear from our crystal structures that this hydrophobic patch plays a role in the formation of extensive homodimeric crystal contacts or in the interaction with the N-terminal amphipathic helix. Importantly, the structural variability in the β-sheet mediated PAS-PAS interactions observed in our crystals, together with the previous report that the hERG PAS domain is a monomer in solution [5], indicates that these PAS-PAS homodimers are most likely not present in the full-length tetrameric channel. Nevertheless, the strong tendency to form contacts correlates well with the proposal that this hydrophobic region mediates the interaction between the PAS domain and other regions in the channel [5], [19]. It is intriguing to consider that, as observed in other PAS domains [37], [38], the putative regulatory role of the channel domains may involve changes in the β-sheet mediated interaction with the channel.

Another parallel between the channel PAS domains and other PAS domains [10] is that regions that immediately precede or follow the domain have important functional roles. Truncation of the N-terminus of KCNH channels, the ∼20 residues that precede the PAS domain, or mutation of some of these residues has robust effects on the kinetic properties of channel gating. Our crystal structures of the dELK and Δ9-hERG PAS domains and the previously reported NMR structures of the hERG PAS domain [15], [16], [17] have clearly established that a stretch of the N-terminus in KCNH channels forms an amphipathic α-helix. Interestingly, the crystal structures show a conformation, with the helix packed against the hydrophobic patch on the β-sheet, which is different from the one in the NMR structures. Although there is evidence that the N-terminus of hERG PAS interacts with other channel regions [18], [39], the packing arrangement in our structures is reminiscent of what occurs in two other PAS domains: the LOV-HTH DNA binding protein and the LOV2 domain of the blue light sensor phototropin 1. In the LOV-HTH DNA binding protein the N-terminal helix packs on the β-sheet, side-by-side with the C-terminal DNA binding domain [35], and in the LOV2 domain of the blue light sensor phototropin 1 a small N-terminal helical extension packs against the sheet together with a long C-terminal helix [36]. It is therefore conceivable that in some functional state of the full-length channel the N-terminal helix of PAS adopts the conformation seen in the structures of dELK and Δ9-hERG PAS.

An issue we have examined in this study is the existence of potential ligand binding sites in the PAS domains of KCNH channels. The structures we have determined provide a mixed response. First, the mEAG and hERG PAS domains display cavities in the same region where other PAS domains have ligand binding sites. In contrast, dELK does not have a preformed cavity; it shows just small openings (less than 20 Å^3^), which can be considered as defects in the packing of the hydrophobic core of the protein [33]. The volumes of the hERG and mEAG cavities (around 120 Å^3^) are modest relative to the 730 Å^3^ of the binding site that holds FMN in the phototropin-LOV1 domain (PDB code 1N9L) [40], but are within the range of the ligand binding sites of PAS domains in the methyl-accepting chemotaxis protein (2 adjacent cavities, 138 Å^3^; PDB code 2QHK- unpublished) or in the DctB sensor domain (137 Å^3^; PDB code 3E4O) [41]. Moreover, a survey of cavities/pockets in protein structures showed that cavities with volumes larger than 100 Å^3^ are uncommon structural features, amounting to less than 5% of the total number of cavities found [33]. This supports the notion that the cavities observed in mEAG and hERG are not just defects in the core of these proteins.

Second, our crystal structures revealed that the lining of the cavities in the PAS domains from KCNH channels includes very few or no polar residues. This is in accordance with the properties of other orphan receptor PAS domains which also have cavities lined by apolar residues, as revealed by a simple visual inspection of PAS domain structures listed in a review [10]. In contrast, PAS domains structures with bound ligands have cavities lined by a mixture of polar and apolar residues, with the polar residues involved in the coordination of the ligand. Clearly, the presence of polar residues in the core cavities increases the potential for binding small molecules by amplifying the variability in the chemical environment of a binding site.

**Figure 7:**
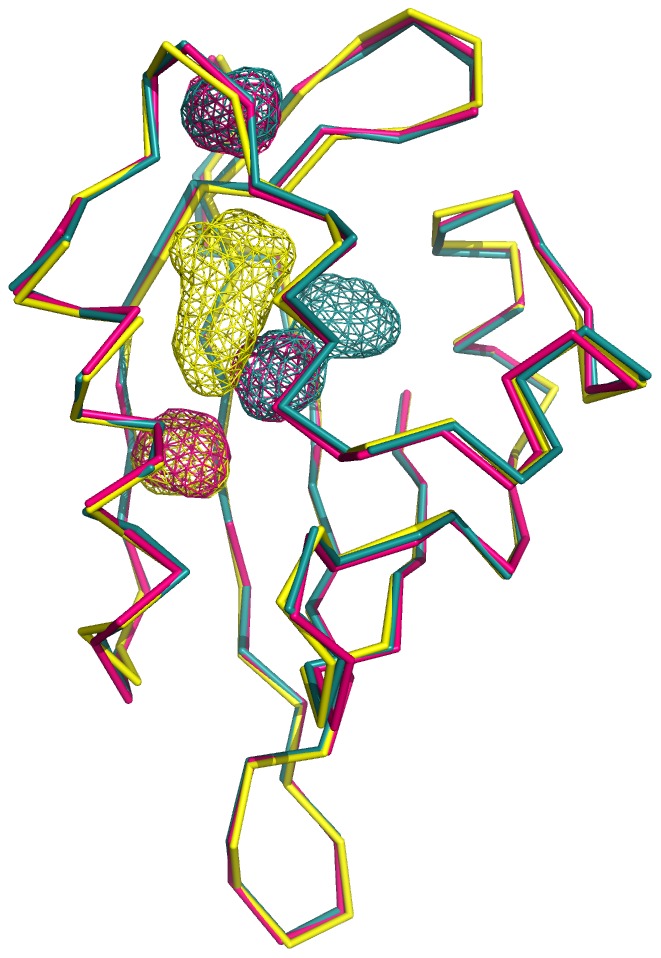
Superposition of three (in red, yellow and cyan Cα trace) of the NMR model structures of the hERG PAS domain (PDB code 1L0W). Cavities detected in the three models are shown as red, yellow or cyan wireframe.

Third, the four molecules in the asymmetric unit of the mEAG PAS domain crystal revealed a variation of up to ∼20% in this domain’s cavity volume which is a reflection of flexibility in the protein’s core through reorientation of side-chains and small adjustments of backbone. Interestingly, the structures of the hERG PAS domain determined by NMR [15], [16], [17] also provide information on the domain’s cavity flexibility. We focused our analysis on the 2L0W structure [16] which has the better statistics. The 20 structures composing the solution ensemble display cavities in the core of the domain which are in many cases coincident with the cavity detected in the X-ray structure (Figure 7). There are also cavities detected in other regions of the domain’s core, in particular in the region where the photoactive yellow protein (a PAS domain) holds its ligand [42].

Overall, our structural results suggest that at room temperature the PAS domains of the KCNH channels sample conformations where the cavities in the core are larger and possibly more chemically varied than seen in our crystal structures. Our data therefore suggests that these channel PAS domains have the potential to bind small molecules, although to date none have been identified.

## Supporting Information

Figure S1 Sequence alignment of PAS domains from hERG, dELK, mEAG and zebra fish ERG (zfERG) channels. Highlighted residues are part of the hydrophobic core of the hERG PAS domain. Residues highlighted in green indicate positions that in dELK, mEAG and zfERG are occupied by polar residues.
(TIF)

Figure S2 Superposition of PAS domain structures from hERG (redbrick) and dELK (blue). Cavity in hERG is shown as redbrick wireframe. Residues in dELK which occupy the space taken by cavity in hERG are shown as blue stick with corresponding atomic Van der Waals volume as a dot representation.
(TIF)

Figure S3 Hydrophobic patch in channel PAS domains. View of the hydrophobic patches on the β-sheets of the PAS domain from the three different channels: a) hERG, b) mEAG and c) dELK. Residues forming patches are shown as stick and are labeled. d) Packing of N-terminal helix against hydrophobic patch in dELK PAS domain. Residues involved in interaction are shown as stick.
(TIF)
