# Supplementary figures and images for: Structural Properties of PAS Domains from the KCNH Potassium Channels
Source: PLoS One. 2013 Mar 15;8(3):e59265. doi: 10.1371/journal.pone.0059265 (PMC3598652; doi:10.1371/journal.pone.0059265)

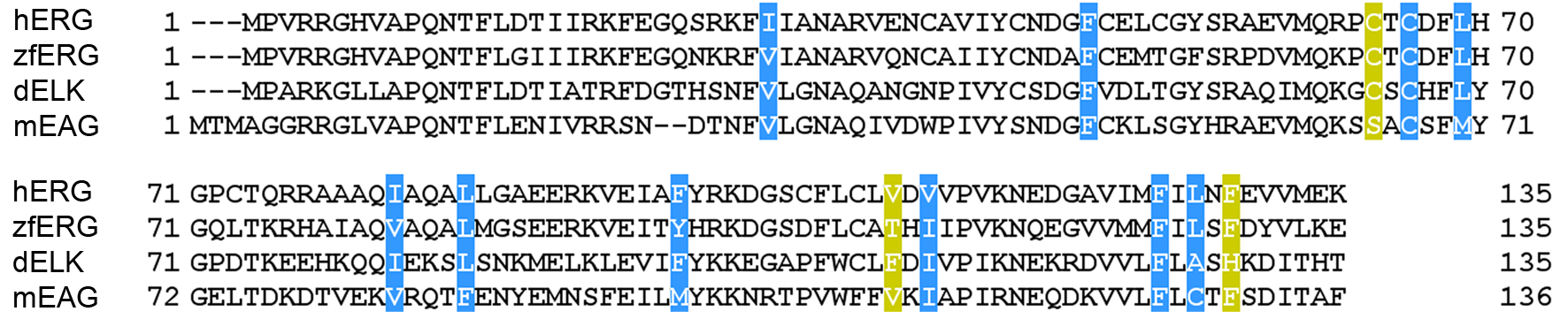

Supplement: Figure S1 — Sequence alignment of PAS domains from hERG, dELK, mEAG and zebra fish ERG (zfERG) channels. Highlighted residues are part of the hydrophobic core of the hERG PAS domain. Residues highlighted in green indicate positions that in dELK, mEAG and zfERG are occupied by polar residues. (TIF) [file pone.0059265.s001.tif]

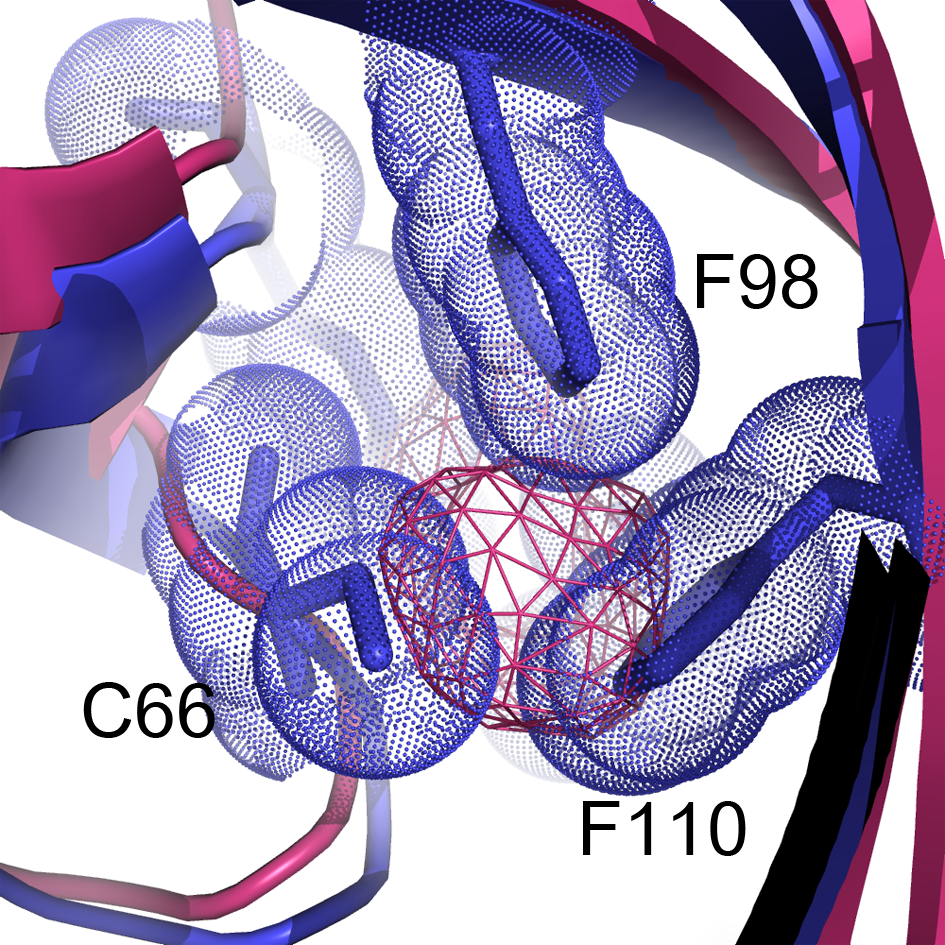

Supplement: Figure S2 — Superposition of PAS domain structures from hERG (redbrick) and dELK (blue). Cavity in hERG is shown as redbrick wireframe. Residues in dELK which occupy the space taken by cavity in hERG are shown as blue stick with corresponding atomic Van der Waals volume as a dot representation. (TIF) [file pone.0059265.s002.tif]

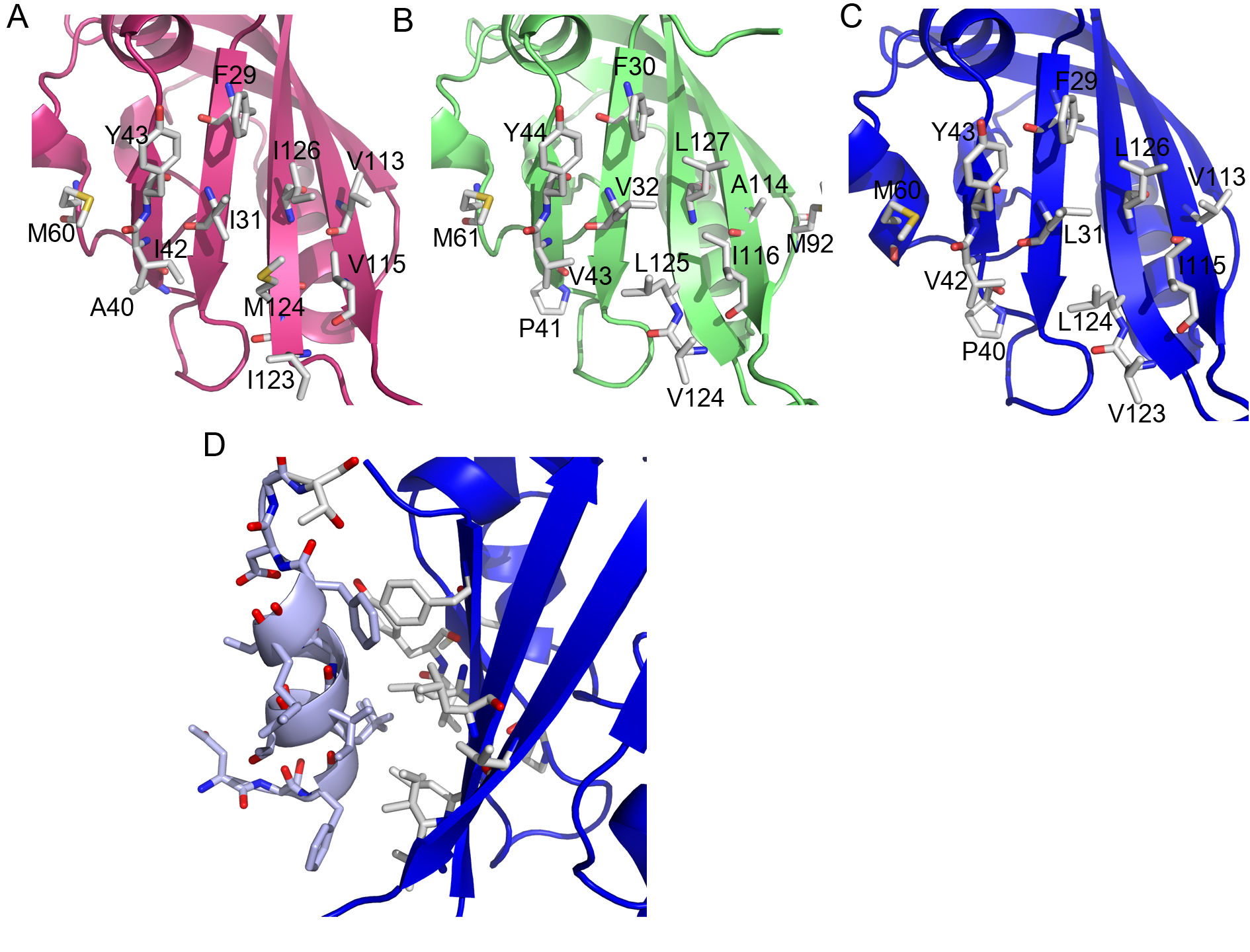

Supplement: Figure S3 — Hydrophobic patch in channel PAS domains. View of the hydrophobic patches on the β-sheets of the PAS domain from the three different channels: a) hERG, b) mEAG and c) dELK. Residues forming patches are shown as stick and are labeled. d) Packing of N-terminal helix against hydrophobic patch in dELK PAS domain. Residues involved in interaction are shown as stick. (TIF) [file pone.0059265.s003.tif]
